# Fetuin-A: A Novel Biomarker of Bone Damage in Early Axial Spondyloarthritis. Results of an Interim Analysis of the SPACE Study

**DOI:** 10.3390/ijms24043203

**Published:** 2023-02-06

**Authors:** Marta Favero, Francesca Ometto, Elisa Belluzzi, Giacomo Cozzi, Laura Scagnellato, Francesca Oliviero, Pietro Ruggieri, Andrea Doria, Mariagrazia Lorenzin, Roberta Ramonda

**Affiliations:** 1 Rheumatology Unit, Department of Medicine-DIMED, Padova University Hospital, 35128 Padova, Italy; 2 Internal Medicine I, Cà Foncello Hospital, 31100 Treviso, Italy; 3 Musculoskeletal Pathology and Oncology Laboratory, Department of Surgery, Oncology and Gastroenterology (DiSCOG), University of Padova, 35128 Padova, Italy; 4 Orthopedics and Orthopedic Oncology, Department of Surgery, Oncology and Gastroenterology, University of Padova, Via Giustiniani 3, 35128 Padova, Italy

**Keywords:** fetuin-A, biomarkers, axial spondyloarthritis, imaging, sacroiliac joint bone damage

## Abstract

Our study aimed to evaluate the association between fetuin-A levels and the presence of radiographic sacroiliitis and syndesmophytes in patients with early axial spondyloarthritis (axSpA) and to identify potential predictors of radiographic damage in the sacroiliac joints (SIJs) after 24 months. Patients diagnosed with axSpA in the Italian cohort of the SpondyloArthritis-Caught-Early (SPACE) study were included. Physical examinations, laboratory tests (including fetuin-A), SIJ,+ and spinal X-rays and MRIs at T0 (diagnosis) and at T24 were considered. Radiographic damage in the SIJs was defined according to the modified New York criteria (mNY). Fifty-seven patients were included in this analysis (41.2% male, median (interquartile range), chronic back pain [CBP] duration of 12 (8–18) months). Fetuin-A levels were significantly lower in patients with radiographic sacroiliitis compared to those without at T0 (207.9 (181.7–215.9) vs. 239.9 (217.9–286.9), respectively, *p* < 0.001) and at T24 (207.6 (182.5–246.5) vs. 261.1 (210.2–286.6) µg/mL, *p* = 0.03). At T0, fetuin-A levels were significantly higher in non-smokers, in patients with heel enthesitis and in those with a family history of axSpA; fetuin-A levels at T24 were higher in females, in patients with higher ESR or CRP at T0 and in those with radiographic sacroiliitis at T0. Fetuin-A levels at T0 were independently negatively associated with the likelihood of radiographic sacroiliitis (OR = 0.9 per 10-unit increase (95% CI 0.8, 0.999), *p* = 0.048); but not with the presence of syndesmophytes. After adjustment for confounders, fetuin-A levels at T0 and T24 were also negatively associated with mNY at T0 (β −0.5, *p* < 0.001) and at T24 (β −0.3, *p* < 0.001), respectively. Among other variables at T0, fetuin-A levels did not achieve statistical significance in predicting mNY at T24. Fetuin-A levels were negatively associated with radiographic damage of the SIJs, but not of the spine, in early axSpA and after 2 years of follow-up. Our findings suggest that fetuin-A levels may serve as a biomarker to identify patients with a higher risk of developing severe disease and early structural damage.

## 1. Introduction

Spondyloarthritis (SpA) is a group of chronic inflammatory rheumatic diseases sharing overlapping features, including axial SpA (axSpA) and peripheral SpA. AxSpA affects mainly the axial skeleton and comprises both patients with radiographic (r-axSpA) structural damage in the sacroiliac joints (SIJs) or in the spine and patients with no structural lesions evident on X-rays (non-radiographic, nr-axSpA) [1,2]. Structural damage on X-rays appears in advanced stages of the disease, thus nr-axSpA may be considered an earlier stage of the disease or a milder disease which may never progress to structural radiographic damage [2]. In nr-axSpA, structural lesions are evident on magnetic resonance imaging (MRI), which is pivotal for early diagnosis in patients with suspected axSpA [3]. MRI detects inflammatory lesions of the bone (i.e., bone marrow edema, BME) which are evident also in the early stages of the disease [2]. In fact, BME has been reported in over 50% of the patients with early axSpA: 51% in spine-MRI and 56.7% in SIJ-MRI. Interestingly, 15% of patients with spine-MRI BME have no lesions on SIJ-MRI [4,5]. It bears noting that early diagnosis of axSpA remains challenging as MRI inflammatory abnormalities may also be found in healthy people, such as runners and post-partum women [6,7]. In this context, basic research could help identify novel biomarkers useful for the early diagnosis of axSpA [6,8,9]. Several molecules have been proposed to date as possible biomarkers for early axSpA with poor results [6,10]. Tumor necrosis factor-alpha (TNF-alpha), interleukin-17 (IL-17), and IL-23, which are key molecules in the pathogenesis of axSpA, have shown a limited association with disease characteristics [6,11,12].

Fetuin-A is a glycoprotein produced by the liver and released into the bloodstream [13]. It is involved in several different biological processes such as the regulation of bone and calcium metabolism and the insulin signaling pathway [13]. Moreover, it has several other functions such as protease inhibitor, atherogenic, and adipogenic factors [13]. It has also a complex role in inflammation. Indeed, it has been reported that fetuin-A has both anti-inflammatory and inflammatory activity depending on the stimulus in different clinical conditions [13,14].

Moreover, fetuin-A is a plasma carrier protein for calcium and phosphate, regulating their levels and inhibiting ectopic calcification [13,15]. It has been suggested to be a potential biomarker for several diseases (i.e., depression in the elderly, coronary artery disease, metabolic syndrome, aortic valve stenosis, rheumatoid arthritis, and type 2 diabetes mellitus risk) [16,17,18,19,20,21]. Fetuin-A has been suggested to have a role in the pathogenesis of axSpA [22,23,24,25] although its association with disease activity and structural damage has not been clarified [26]. Sari et al. found increased fetuin-A levels in patients with ankylosing spondylitis regardless of disease activity and treatment [24]. Harman et al. observed an increase in fetuin-A levels in patients with axSpA compared to healthy subjects [22], whereas Przepiera-Będzak et al. detected a decrease in fetuin-A levels in axSpA compared to controls [23]. Tuylu et al. found that patients with syndesmophytes had significantly higher levels of fetuin-A compared to patients without syndesmophytes [25]. In addition, a recent study demonstrated that patients with axSpA and inflammatory bowel disease had lower serum levels of fetuin-A compared to controls [27]. The molecular mechanisms involved in bone alterations in axSpA are poorly understood especially due to the difficulty in retrieving spinal tissues in these patients [28]. Some preliminary evidence suggests that fetuin-A may be associated with new bone formation and thus to bony fusion of SIJs, syndesmophytes, and enthesophytes, which are characteristic of advanced axSpA [28].

The aim of this study was to evaluate the association between fetuin-A levels with the presence of radiographic sacroiliitis and syndesmophytes in patients with early axSpA included in the SPACE study. Secondary objectives of the study were to evaluate the association between fetuin-A and radiographic damage in the SIJs (defined according to the modified New York (mNY) criteria) and to identify potential predictors of radiographic damage in the SIJs after 24 months.

## 2. Results

### 2.1. Characteristic of the Patients in the Cohort (Descriptive Analysis)

Fifty-seven patients were included in this analysis, 41 patients had complete data at T24. Characteristics of the patients are reported in Table 1, variables at T0 and T24 are reported in Table S1. Males were 24 (42.1%) and the median (interquartile range) age at chronic back pain (CBP) onset was 28 (22–36) years. Patients had a median CBP duration of 12 (8–18) months.

Continuous variables are expressed as mean ± standard deviation or median and interquartile range, as appropriate, and categorical variables as number (%). Data were compared between the groups with Mann-Whitney U test and Chi-square or Fisherman’s exact test.

CBP = chronic back pain, HLA = human leukocyte antigen, IBD = inflammatory bowel disease, NSAIDs = non-steroidal anti-inflammatory drugs, CRP = C reactive protein, ESR = erythrocytes sedimentation rate, BASDAI = Bath Ankylosing Spondylitis Disease Activity Index, BASFI = Bath Ankylosing Spondylitis Functional Index, BASMI = Bath Ankylosing Spondylitis Metrology Index, ASDAS = Ankylosing Spondylitis Disease Activity Score, HAQ = health assessment questionnaire, MASES = Maastricht Ankylosing Spondylitis Enthesitis Score, VAS = visual analogue scale, mNY = modified criteria of New York score, mSASSS = modified Stoke Ankylosing Spondylitis Spinal Score, SPARCC = Canadian Spondyloarthritis Research Consortium, SIJ = sacroiliac joint.

Continuous variables are expressed as mean ± standard deviation and categorical variables as number (%). Data were compared between the groups with Mann-Whitney U test and Chi-square or Fisherman’s exact test.

CBP = Chronic back pain, HLA = human leukocyte antigen, IBD = inflammatory bowel disease, NSAIDs = non-steroidal anti-inflammatory drugs, CRP = C reactive protein, ESR = erythrocytes sedimentation rate, BASDAI = Bath Ankylosing Spondylitis Disease Activity Index, BASFI = Bath Ankylosing Spondylitis Functional Index, BASMI = Bath Ankylosing Spondylitis Metrology Index, ASDAS = Ankylosing Spondylitis Disease Activity Score, HAQ = health assessment questionnaire, MASES = Maastricht Ankylosing Spondylitis Enthesitis Score, VAS = visual analogic scale, mNY = modified criteria of New York score, mSASSS = modified Stoke Ankylosing Spondylitis Spinal Score, SPARCC = Canadian Spondyloarthritis Research Consortium, SIJ = sacroiliac joint.

Characteristics of the patients in the tree cohorts of the SPACE study are reported in Table S2.

### 2.2. Levels of Fetuin-A in the Cohort (Descriptive Analysis)

Levels of fetuin-A were 222.8 (203.3–251) at T0 and 239.7 (199–275.1) µg/mL at T24 in the entire cohort (*p* = 0.104). Fetuin-A levels at T0 and T24 are reported according to the categorical variables in Table S3, and the correlation between fetuin-A levels and continuous variables is reported in Table S4.

**Figure 1:**
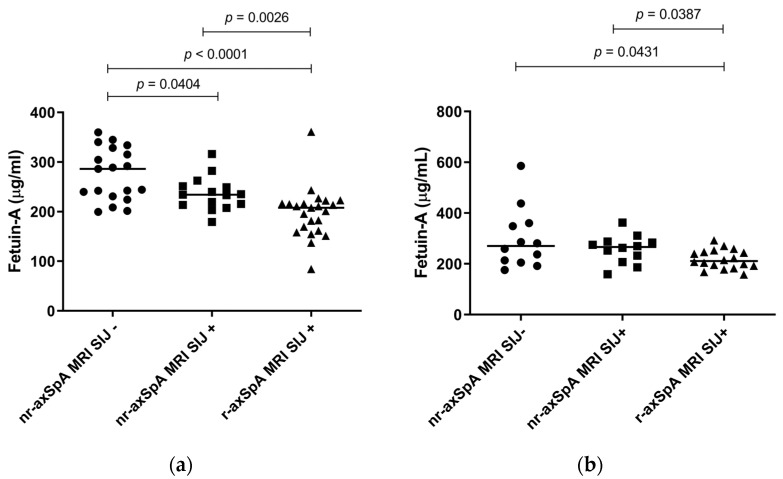
Association of fetuin-A at T0 (**a**) and at T24 (**b**).

Levels of fetuin-A at T0 were higher in nr-axSpA MRI SIJ- compared to nr-axSpA MRI SIJ+ and also compared to r-axSpA MRI SIJ+; levels were also higher in nr-axSpA MRI SIJ+ compared to r-axSpA MRI SIJ+ (Figure 1). At T24 a trend toward higher fetuin-A levels in patients with no signs of SIJ involvement compared to the other patients was observed but the difference was significant only in nr-axSpA MRI SIJ- vs. r-axSpA MRI SIJ+ and nr-axSpA MRI SIJ+ vs. r-axSpA MRI SIJ+.

Fetuin-A levels at T0 and T24 were positively associated (r = 0.3, *p* = 0.06) (Table S4).

At T0, fetuin-A was significantly higher in non-smokers compared with smokers (237.1 (210.8–288.8) vs. 213.4 (182.1–223.1), respectively, *p* = 0.01); in patients with heel enthesitis compared with those without (232 (208.6–262.4) vs. 207.4 (182.1–214.8), respectively, *p* = 0.03) and with a family history of SpA compared with no family history (234.7 (215.5–287.6) vs. 211 (199.3–242.6), respectively *p* = 0.04). Fetuin-A T0 levels were significantly lower in patients with radiographic sacroiliitis compared to those with no sacroiliitis: 207.9 (181.7–215.9) vs. 239.9 (216.4–287.6), respectively, *p* < 0.001 (Table S3). Also, fetuin-A T0 levels negatively correlated with mNY at T0 (r = −0.59, *p* < 0.001) and SPARCC spine at T0 (r = −0.31, *p* = 0.02) (Table S4).

At T24, fetuin-A was significantly lower in males (males 204.7 (180.1–245) vs. females 262.9 (232.5–285.7), *p* < 0.001), in patients who had elevated CRP or ESR at T0 (232.5 (186.5–259.2) vs. 261.1 (211.2–317), *p* = 0.04), and in patients with radiographic sacroiliitis at T0 (207.6 (182.5–246.5) in those with sacroiliitis vs. 261.1 (210.2–286.6) in those without, *p* = 0.03) (Table S3). Fetuin-A levels at T24 correlated with the age at CBP onset (r = 0.42, *p* = 0.01) (Table S4) and negatively correlated with indices of radiographic damage both at T0 (mNY: r = −0.38, *p* = 0.01; SPARCC SIJ: r = −0.33, *p* = 0.04, SPARCC spine: r = −0.31, *p* = 0.05) and at T24 (mNY: r = −0.39, *p* = 0.01) (Table S4).

### 2.3. Factors Associated with Radiographic Sacroiliitis at T0 (Univariate Analysis and Multivariate Logistic Regression)

Characteristics of the patients according to the presence of radiographic sacroiliitis are reported in Table 1 together with results of univariate tests. Twenty-one patients in the cohort (38.8%) presented radiographic sacroiliitis at T0. Patients with radiographic sacroiliitis had lower fetuin-A levels (*p* < 0.001), BASDAI (*p* = 0.03) and MASES (*p* = 0.04) at T0. Heel enthesitis was less frequent in patients with radiographic sacroiliitis at T0 (*p* = 0.04) (Table 1).

Variables included in the multivariate logistic regression analysis were male sex, smoking, heel enthesitis at T0, fetuin-A levels at T0, BASDAI, and MASES at T0. No variable was excluded because of collinearity. The regression model was statistically significant, X^2^ (df = 6, N = 57) 15.61, *p* = 0.016 (Table 2). The model explained 32.7% of the variance in radiographic sacroiliitis and correctly classified 63.2% of cases. Only increasing fetuin-A levels at T0 were significantly associated with a reduction in the likelihood of radiographic sacroiliitis: OR = 0.9 per 10-unit increase (95% CI 0.8, 0.999), *p* = 0.048.

### 2.4. Factors Associated with Syndesmophytes at T0 (Univariate Analysis and Multivariate Logistic Regression)

Characteristics of the patients according to the presence of syndesmophytes are reported in Table 1 together with results of univariate tests. Six (10.5%) patients presented syndesmophytes at T0. Subjects with syndesmophytes had an older age of onset of CBP (*p* = 0.01), higher BMI (*p* = 0.05) and mSASSS (*p* < 0.001) compared to those without. Male sex was significantly more frequent in patients with syndesmophytes (*p* = 0.03). Levels of fetuin-A were not significantly different in patients with syndesmophytes and those without: 209.8 (203.3–249.3) versus 224.1 (204.4–256.7) µg/mL, respectively, *p* = 0.68.

Variables included in the multivariate logistic regression analysis were male sex, BMI, and age at CBP onset. No variable was excluded because of collinearity. The regression model was statistically significant, X^2^ (df = 3, N = 57) 22.02, *p* < 0.001 (Table 2). The model explained 64.5% of the variance in radiographic syndesmophytes and correctly classified 89.5% of cases. Males were more likely to have syndesmophytes: OR 90.48 (95% CI 1.5; 5644.9), *p* = 0.033. Also, increasing age at CBP onset was significantly associated with an increase in the likelihood of syndesmophytes: OR = 1.28 per year increase, 95% (CI 1.04; 1.6), *p* = 0.019.

### 2.5. Factors Associated with Radiographic Damage in the SIJs (mNY) at T0 and at T24 (Univariate Analysis and Multivariate Linear Regression)

Associations of mNY at T0 and T24 with categorical variables are reported in Table S5, whereas association with continuous variables is reported in Table S6.

**Figure 2:**
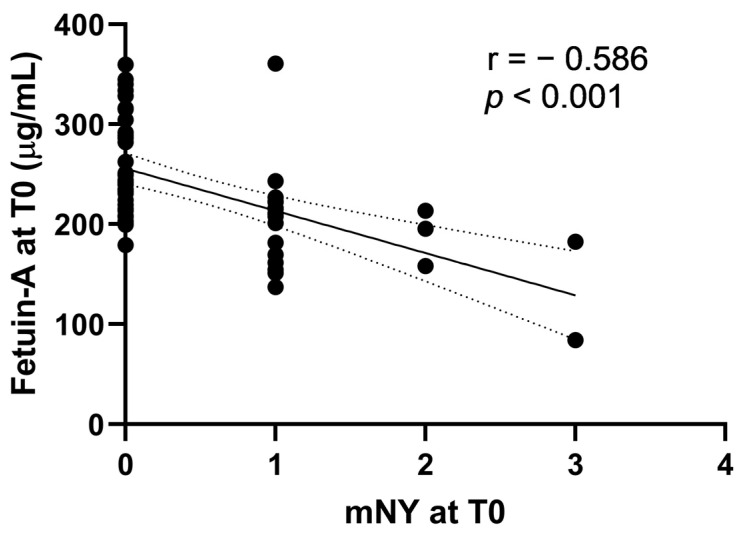
Scatterplot: Association of Fetuin-A at T0 and of mNY at T0.

mNY at T0 correlated significantly with fetuin-A levels at T0 (*p* < 0.001) (Figure 2), and, as expected, with SPARCC spine (*p* = 0.02) and SPARCC SIJ (*p* < 0.001). Multiple linear regression analysis was computed to determine what factors at T0 were significantly associated with mNY at T0. Variables included were male sex, uveitis, BASDAI, and fetuin-A at T0. No variable was excluded because of collinearity. The regression model was statistically significant, R^2^ = 0.37, F (4) = 7.45; *p* < 0.001 (Table 3). Only fetuin-A levels at T0 were significantly associated with mNY at T0 (β −0.5, *p* < 0.001).

**Figure 3:**
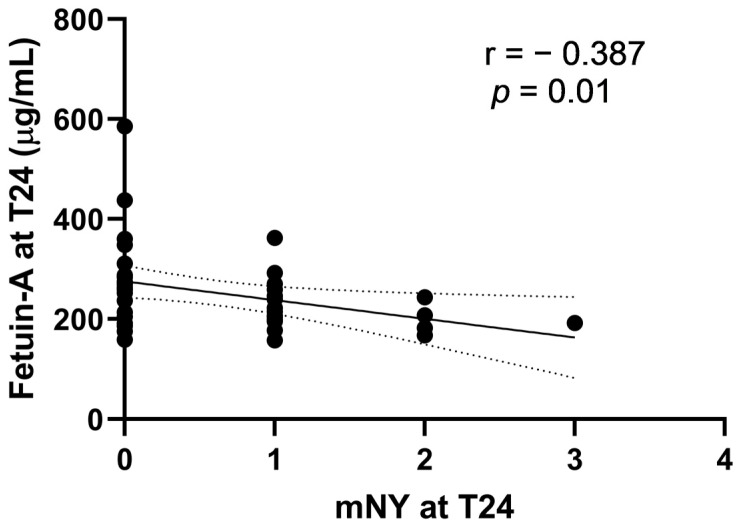
Scatterplot: Association of Fetuin-A at T24 and of mNY at T24.

mNY at T24 correlated significantly with fetuin-A levels at T24 (*p* < 0.001) (Figure 3) and BASDAI at T24 (*p* = 0.07) and, as expected, with SPARCC spine (*p* = 0.07) and SPARCC SIJ (*p* = 0.01) (Table S6). Multiple linear regression analysis was run to determine what factors at T24 were significantly associated with mNY at T24. Variables included in the analysis were BASDAI and fetuin-A at T24. No variable was excluded because of collinearity. The regression model was statistically significant, R^2^ = 0.16, F (2) = 3.51; *p* = 0.40 (Table 3).

Again, only fetuin-A levels at T24 were significantly associated with mNY at T24 (β −0.3, *p* < 0.001).

### 2.6. Baseline Predictors of Radiographic Damage in the SIJs (mNY T24) (Univariate Analysis and Multivariate Linear Regression)

**Figure 4:**
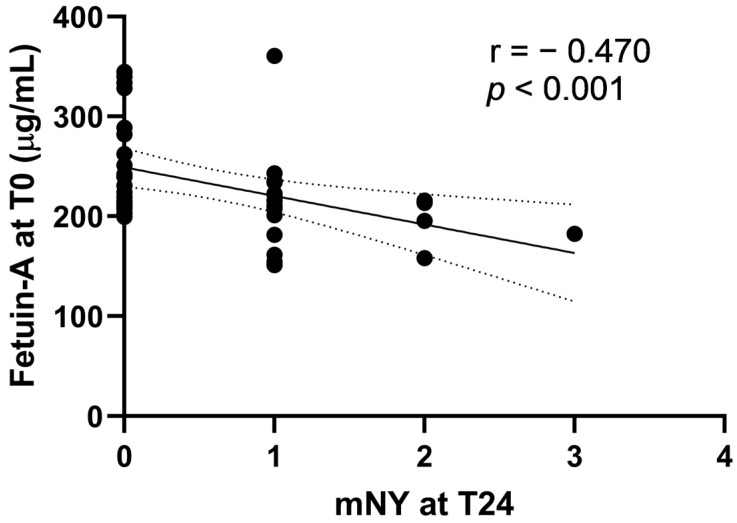
Scatterplot: Association of Fetuin-A at T0 and of mNY at T24.

Variables at T0 associated with mNY at T24 were fetuin-A levels (*p* < 0.001) (Figure 4), BASDAI at T0 (*p* < 0.001), VAS pain (*p* = 0.01), VAS disease activity (*p* = 0.01), mNY (*p* < 0.001), and SPARCC SIJ (*p* < 0.001) (Table S6). A multiple linear regression model was used to test if variables at T0 predicted mNY at T24. Variables included in the analysis were radiographic sacroiliitis, SPARCC spine, SPARCC SIJ, mNY, ASDAS, BASDAI, VAS pain, VAS disease activity, and fetuin-A at T0. Variables excluded because of collinearity were radiographic sacroiliitis, SPARCC spine, SPARCC SIJ (collinear with mNY at T0), ASDAS, VAS pain, and VAS disease activity (collinear with BASDAI at T0). The regression model was statistically significant, R^2^ = 0.85; F(5) = 40.69; *p* < 0.00 (Table 3). Only mNY at T0 was significantly associated with mNY at T24 (β 0.9, *p* < 0.001). Fetuin-A levels did not achieve statistical significance in the model.

## 3. Discussion

In our study fetuin-A levels appeared to be lowered in subjects with radiographic damage in the SIJs. The association is evident in early diagnosed axSpA patients and persists after 2 years. Low levels of fetuin-A are not associated with radiographic damage in the spine and, although low fetuin-A levels at diagnosis are more frequently observed in those with higher radiographic damage in the SIJs at 24 months, this association is not significantly predictive of further damage [26]. Notably, an inverse relationship between fetuin-A levels and MRI findings was also observed [26].

The SPACE cohort provides the perfect opportunity to study the trend of fetuin-A levels in the early phase of axSpA. Fetuin-A levels appear to be lower in smokers, in subjects with heel enthesitis, and in those with a family history of axSpA; furthermore, levels are also lower in males, although only at 24 months. Although fetuin-A levels decreased over time in the entire cohort, the difference was not significant.

In our study, SIJ damage at baseline was associated only with fetuin-A levels after adjustment for confounders, whereas the presence of syndesmophytes was associated with male sex and the age of CBP onset, as previously reported [8,12,29].

Low fetuin-A levels at diagnosis were the only factor independently associated with the presence of radiographic sacroiliitis. Fetuin-A levels also correlated inversely with the extent of the radiographic damage at baseline (measured with mNY), and the association was the most significant compared to other clinical measures, including BASDAI. Furthermore, this association was also observed 24 months after diagnosis, whereas no other clinical or laboratory measures were associated independently with the radiographic damage. These results suggest that fetuin-A levels may serve as a predictive biomarker for patients who may develop a more severe disease and structural damage, possibly with a higher sensitivity than CRP, which is currently the only available biomarker in axSpA monitoring. Besides the correlation with SIJ damage, fetuin-A levels are not independently associated with the presence of syndesmophytes, although a downtrend was observed. Older age at CBP onset and male sex were associated with a higher frequency of syndesmophytes. The lack of association between fetuin-A levels and radiographic damage in the spine may be attributed to the early disease of the patients in our cohort. SIJs involvement usually occurs earlier than the spinal involvement [30]; in fact, only 10% of patients in the study presented with syndesmophytes vs. 40% with radiographic sacroiliitis. Tuluy et al. reported that higher fetuin-A levels were associated with the presence of syndesmophytes, and the study was conducted in a cohort of patients with longstanding disease [24].

Notwithstanding the lack of association between fetuin-A levels and MRI scores both in the SIJs and in the spine, in our study, we found lower fetuin-A levels in patients with SIJ inflammation on MRI compared to those without, reinforcing the hypothesis that fetuin-A may be reduced in the case of an ongoing inflammatory process in the bone. Fetuin-A is an inhibitor of mineralization and low levels of fetuin-A have been correlated with vascular calcification and increased cardiovascular diseases [31]. In addition, high levels of synovial fluid fetuin-A have been associated with the presence of calcium crystal in osteoarthritis [32]. In view of this evidence, we hypothesized that fetuin-A may be depleted during the early stages of spondylarthritis since it acts as an inhibitor of the bone formation.

To date, few studies in the literature have focused on fetuin-A in axSpa. Unfortunately, assays are not standardized, and the study cohorts are not comparable due to broadly different socio-demographic characteristics and clinical features. Most studies were conducted in patients with longstanding disease, with a disease duration ranging from 10 to 20 years [22,24,25,27]. Furthermore, only two studies considered structural damage [22,25] and only one in the SIJs [25]. Although Harman et al. found no association between fetuin-A levels and SIJ X-rays, they reported a correlation between fetuin-A levels and the Bath Ankylosing Spondylitis Radiology Index: a combined score that evaluates SIJs, cervical and lumbar spine and hip radiographic changes [22].

Furthermore, a few studies report an association between fetuin-A levels and bone metabolism but do not allow the drawing of definite conclusions on its role. Fetuin-A seems to be associated with reduced bone catabolism (with an inverse relationship with C-telopeptide) in type 2 diabetes [33], while it is reported to be associated with increased osteoporosis in postmenopausal women [34]. The SPACE study did not include bone metabolism evaluation, which is intriguing given the increased risk of osteoporosis in axSpA patients. Further analysis should investigate the potential association of fetuin-A in bone formation and catabolism especially in these patients.

Overall, the available literature shows that fetuin-A levels are increased in patients with established axSpA patients vs. healthy controls [22,24], except for the study by Przepiera-Będzak et al. which showed lower levels in subjects with a 10-year disease duration compared to controls [27]. The same authors also found reduced levels of fetuin-A in patients with earlier axSpA vs. healthy subjects, with an inverse correlation with CRP levels and a positive correlation with VEGF [23], thus corroborating our findings that fetuin-A levels may be reduced in those with active disease. We also assessed fetuin-A levels as a potential predictor of radiographic damage 24 months after diagnosis. Although we observed a significant negative correlation with the mNY score at 24 months in univariate analysis, after adjustment for confounders, baseline fetuin-A levels were not a significant predictor of radiographic progression. Independent baseline predictors of radiographic progression at 24 months were the presence of a radiographic sacroiliitis and a higher BASDAI.

Importantly, this is the first study evaluating the role of fetuin-A in a cohort of patient affected by early axSpA (inflammatory low back pain <2 years) well characterized from both a clinical and a radiographic (x-ray and MRI) point of view. We would be remiss not to mention some of the limitations of our study. Firstly, fetuin-A levels were not measured in a group of heathy controls matched for age and sex, even though our patients had an early disease with low back pain onset between 3 and 24 months. Secondly, we had available data on smoking status and BMI, but not other cardiovascular diseases such as hypertension and dyslipidemia or kidney disease, which may influence the fetuin-A levels. Thirdly, although patients were not undergoing treatment at the time of enrollment, fetuin-A levels at 24 months may have been influenced by therapies initiated thereafter. It bears noting, however, that Sari et al. did not find any association between fetuin-A levels and treatment [24]. Similarly, Harman et al. reported that fetuin-A levels were not affected by the anti-TNF treatment [22]. Our patients were treated according to the standard of care towards clinical remission, and different treatments were homogeneously distributed among groups. Importantly, we were able to confirm at 24 months data that were already found at the baseline, when any treatment was started.

## 4. Materials and Methods

### 4.1. Study Population, Clinical Assessment and Radiographic Evaluation

Patients included in this study are subjects with a diagnosis of axSpA in the Italian cohort of the SpondyloArthritis-Caught-Early (SPACE) study, which is an ongoing observational cohort study [4,12]. Patients were enrolled in the study after providing written informed consent. The Local Ethical Committee approved the study protocol (no. 2438P), and the study was carried out in accordance with the principles of the Declaration of Helsinki.

Inclusion criteria of the SPACE study were patients with chronic back pain (CBP; duration ≥3 months and ≤2 years; age of onset <45 years) were recruited from six different rheumatology outpatient clinics in the Netherlands (Amsterdam, Gouda, Leiden), Norway (Oslo), Sweden (multiple sites), and Italy (Padua). Only patients from the Italian center were considered for the present study. At enrolment, all patients underwent a full diagnostic workup including physical examinations, SIJs, and spinal plain radiographs and MRIs, following a standardized protocol [5], to establish a diagnosis of axSpA. A detailed description of the clinical and radiographic assessment of the SPACE cohort has previously been published [4,5,12]. In the SPACE study, subjects are followed prospectively and undergo clinical examinations at T0 (diagnosis) and at every 6 months thereafter. Radiographic examinations (radiographs and MRI) were performed at T0 and at 12 and 24 months for follow-up. In this study, data at T0 and T24 were considered. During the follow-up, patients were treated according the usual clinical practice with a treat-to-target approach [35] in order to achieve ASDAS-CRP remission [36,37]. Included treatments were non-steroidal anti-inflammatory drugs (NSAIDs) and synthetic or biological disease modifying anti-rheumatic drugs according to EULAR guidelines [38]. Data considered in this analysis were demographic characteristics, history of inflammatory low back pain, smoking status, comorbidity, body mass index (BMI), and human leukocyte antigen (HLA)-B27 positivity. Laboratory tests included reactive protein C (CRP) and erythrocyte sedimentation rate (ESR). Clinical examination included evaluation of the spine, SIJs, tender/swollen joint count, dactylitis, tender entheseal points assessed with by Maastricht Ankylosing Spondylitis Enthesitis Score (MASES) [39], and Bath Ankylosing Spondylitis Measure Index (BASMI) [40]. Questionnaires and composite indexes were also collected: health assessment questionnaire (HAQ) for Spondyloarthritis, visual analogic scale (VAS)-pain (0-10), VAS-disease activity, the VAS night pain scale, the Bath Ankylosing Spondylitis Disease Activity Index (BASDAI) [41], the Bath Ankylosing Spondylitis Functional Index (BASFI) [42], and Ankylosing Spondylitis Disease Activity Score (ASDAS)-CRP.

Radiographic measures included mNY criteria [43] and modified Stoke Ankylosing Spondylitis Spine Score (mSASSS) [44] for scoring, respectively, SIJs and spine bone changes on radiographs, and the Spondyloarthritis Research Consortium of Canada (SPARCC) scoring system for grading inflammatory lesions on MRI (both for SIJs and spine) [45]. Presence of radiographic sacroiliitis was defined by an mNY score ≥1; presence of syndesmophytes was read by two expert radiologists. A detailed description of the clinical and radiographic assessment of the SPACE cohort has previously been published [4,5,12]. At T0, axSpA patients in the SPACE study were divided into three cohorts on the basis of radiographic and MRI examination: nr-axSpA patients without signs of sacroiliitis on MRI (nr-axSpA MRI SIJ-), nr-axSpA patients with signs of sacroiliitis on MRI (nr-axSpA MRI SIJ+), and patients with radiographic signs of sacroiliitis (r-axSpA MRI SIJ) [4,5]. No control group was established for this study.

### 4.2. Fetuin-A testing

Blood samples were collected at the T0 and T24 visits without overnight fasting. Serum samples were stored at −80 °C until they were analyzed. Serum fetuin-A levels were tested using a commercial kit available from Biovendor (Brno, Czech Republic) following the manufacturer’s protocol. Absorbance was read using iMark™ Microplate Absorbance Reader (Bio-rad Laboratories inc., Hercules, CA, USA).

### 4.3. Statistical Analysis

Characteristics of patients are described in the entire study cohort and according to the presence at T0 of radiographic sacroiliitis and syndesmophytes. Normal distributions of continuous variables were tested using the Shapiro-Wilk test and, if normality was satisfied, the data were shown as means ± standard deviations (SD); variables with a non-normal distribution were presented as medians with the corresponding interquartile range. No variable was normally distributed among the categories, only non-parametric tests were used. To compare the characteristics of the three cohorts of patients (nr-axSpA MRI SIJ−, nr-axSpA MRI SIJ+, r-axSpA MRI SIJ+) Kruskal-Wallis test for continuous variables, and Chi square or Fisher’s exact test for categorical variables were used. The association between radiographic sacroiliitis and syndesmophytes with baseline characteristics of the patients was tested using the Mann-Whitney-U test or T-test for continuous variables, and Chi square or Fisher’s exact test for categorical variables, as appropriate. Variables achieving a *p* < 0.10 in univariate analysis were then included in a multivariate logistic regression model with radiographic sacroiliitis or syndesmophytes as an outcome. Results are expressed in terms of odds ratio (OR) and 95% confidence interval (CI).

Radiographic damage in the SIJs was expressed as mNY score at T0 and T24. The association between radiographic damage and characteristics of the patients at each time point was tested with Spearman’s correlation for continuous variables and Mann-Whitney-U test or T-test for categorical variables. Variables achieving a *p* < 0.10 in univariate analysis were then included in a multivariate linear regression model with mNY score at T0 and T24 as the outcome. Results are expressed in terms of B coefficient and its 95% CI and β coefficient.

Likewise, to identify predictors of radiographic damage, the association between mNY score at T24 and characteristics of the patients at T0 was tested, as above. Variables achieving a *p* < 0.10 in univariate analysis were included in a multivariate linear regression model with mNY score at T24 as the outcome.

In multivariable regression analyses, collinearity was assessed by the variance inflation factor (VIF) adopting a cut off = 2 as an exclusion criterion. Significant values were those achieving a *p* value ≤ 0.05. Statistical analysis was performed with IBM Corp. Released 2017. IBM SPSS Statistics for Windows, Version 25.0. Armonk, NY, USA: IBM Corp.

## 5. Conclusions

In conclusion, our study confirmed the association between fetuin-A levels and SIJ damage at the baseline, indicating that fetuin-A may be a novel early biomarker in axSpA. In addition, this study suggests the role of baseline fetuin-A as a biomarker of structural progression at the SIJ level. Further studies are warranted to confirm our in larger with longer follow-up period to ascertain the trends of fetuin-A levels over time and identify cut-offs of fetuin-A levels which may guide clinicians in the early diagnosis of axSpA.

## Figures and Tables

**Table 1:** Characteristics of the patients in the entire cohort at T0 (*n* = 57), and according to the presence of radiographic sacroiliitis and syndesmophytes, results of univariate analysis.

	All Patients	No Radiographic Sacroiliitis	Radiographic Sacroiliitis	*p* Value	No Syndesmophytes	Syndesmophytes	*p* Value
Number of individuals	57	36	21		51	6	
Male sex	24 (42.1)	12 (33.3)	12 (57.1)	0.08 *	19 (37.3)	5 (83.3)	0.03 **
BMI	23.5 (21.1–26.1)	23.5 (21–26.8)	23.5 (21.1–24.8)	0.92	23.3 (20.9–25.4)	26.3 (23.8–32.3)	0.05 **
Smoking	19 (33.3)	9 (25)	10 (47.6)	0.08 *	16 (31.4)	3 (50)	0.36
Age of onset CBP	28 (22–36)	29.5 (21.8–37.3)	26 (23–32)	0.49	28 (21.5–34)	41 (37–42.8)	0.01 **
Duration CBP	12 (8–18)	12 (9.5–18)	12 (8–20)	0.75	12 (8–18)	15 (12.5–19)	0.25
HLA-B27 positivity	22 (38.6)	12 (33.3)	10 (47.6)	0.29	21 (41.2)	1 (16.7)	0.24
IBP	57 (100)	36 (100)	21 (100)	-	51 (100)	6 (100)	-
Heel enthesitis	46 (80.7)	32 (88.9)	14 (66.7)	0.04 **	40 (78.4)	6 (100)	0.21
Dactilitis	13 (22.8)	7 (19.4)	6 (28.6)	0.43	13 (25.5)	0 (0)	0.16
IBD	8 (14)	5 (13.9)	3 (14.3)	0.97	7 (13.7)	1 (16.7)	0.84
Psoriasis	21 (36.8)	15 (41.7)	6 (28.6)	0.32	17 (33.3)	4 (66.7)	0.11
Peripheral arthritis	24 (42.1)	13 (36.1)	11 (52.4)	0.23	20 (39.2)	4 (66.7)	0.2
Family history	28 (49.1)	19 (52.8)	9 (42.9)	0.47	26 (51)	2 (33.3)	0.41
Response to NSAIDs	55 (96.5)	35 (97.2)	20 (95.2)	0.69	49 (96.1)	6 (100)	0.62
Uveitis	4 (7)	4 (11.1)	0 (0)	0.11	3 (5.9)	1 (16.7)	0.33
Fetuin-A, µg/mL	222.8 (203.3–251)	239.9 (217.9–286.9)	207.9 (181.7–215.9)	<0.001 **	224.1 (204.4–256.7)	209.8 (204.6–239.7)	0.68
Elevated CRP or ESR	30 (52.6)	16 (44.4)	14 (66.7)	0.11	27 (52.9)	3 (50)	0.89
CRP, mg/L	2 (1–5)	2.5 (1–5)	2 (1–5)	0.97	2 (1–5)	3.5 (1.3–5.8)	0.97
ESR mm/h	17.6 ± 15.6	12 (7.8–20)	14 (8–25)	0.87	13 (7.5–20)	16 (9.8–28.3)	0.5
							0.9
BASDAI	4.4 ± 2.5	4.9 (3.2–7.2)	3.1 (1.5–5.2)	0.03 **	4.4 (2.4–6.9)	2.3 (1.1–5.3)	0.2
BASFI	0.8 (0.2–2.3)	0.8 (0.2–2.3)	1.2 (0.3–2.3)	0.19	0.8 (0.2–2.6)	1.2 (0.5–1.7)	0.82
BASMI	0 (0–1)	0 (0–1)	0 (0–1.3)	0.8	0 (0–1)	0 (0–2.3)	0.98
ASDAS	2.5 ± 0.8	2.8 (1.8–3.1)	2.4 (1.8–2.9)	0.6	2.6 (1.9–3.1)	2 (1.3–2.7)	0.18
HAQ	0.1 (0–0.5)	0.2 (0–0.6)	0.1 (0–0.4)	0.38	0.1 (0–0.5)	0.3 (0–0.4)	0.97
MASES	3 (1–5)	4 (1.8–5.3)	3 (1–3)	0.04 **	3 (1–5)	3 (1–5)	0.53
VAS pain	4 (1–6)	4 (2–7)	2 (1–5)	0.13	4 (1.5–6)	2.5 (1–6.3)	0.53
VAS disease activity	3 (1–7)	4 (1.8–7)	2 (1–5)	0.17	4 (1–6.5)	2 (0.3–6)	0.36
Night pain	3 (0–6)	3 (0.8–6.3)	1 (0–6)	0.45	3 (0–6.5)	2 (0.3–3.8)	0.62
Radiographic sacroliliitis	21 (36.8)	0 (0)	21 (100)	-	19 (37.3)	2 (33.3)	0.85
Syndesmophytes	6 (10.5)	4 (11.1)	2 (9.5)	0.85	0 (0)	6 (100)	-
mNY	0 (0–1)	0 (0–0)	1 (1–1)	-	0 (0–1)	0 (0–0.8)	0.61
mSASSS	2 (0–5)	2 (0–5)	3 (2–5)	0.36	2 (0–4)	9.5 (5.8–11.8)	<0.001 **
SPARCC SIJ	2 (0–5)	0 (0–2)	5 (0–9)	0.09 ^†^	0 (0–5)	2.5 (0.5–3.8)	0.89
SPARCC spine	3 (0–16)	1 (0–12)	8 (0–21)	0.09 ^†^	0 (0–1)	0 (0–0.8)	0.63

** Variables achieving a significant association with the outcome (radiographic sacroiliitis or syndesmophytes) with *p* ≤ 0.5; * Variables included in the multivariate analysis as achieving an association with the outcome with *p* < 0.1; ^†^ Variables achieving a significant association with the outcome, not included in the multivariate analysis (expected association between radiographic measures).

**Table 2:** Factors associated with radiographic sacroiliitis and syndesmophytes at T0, results of multivariate logistic regression analysis.

Factors Associated with Radiographic Sacroiliitis at T0		
	OR (95%C.I.)	*p* value
Male sex	1.3 (0.3; 5.6)	0.76
Smoking	1.2 (0.3; 4.6)	0.824
Heel enthesitis at T0	0.4 (0.1; 2)	0.278
BASDAI at T0. per unit increase	0.9 (0.7; 1.2)	0.361
MASES at T0	0.8 (0.6; 1.2)	0.284
Fetuin-A at T0 (µg/mL), per 10-unit increase	0.9 (0.8; 0.999)	0.048
Constant	78.8 (0; 0)	0.032
X^2^ (df = 6, N = 57) 15.61, *p* = 0.016; Nagelkerke R^2^ 32.7% The model classified correctly 63.2% cases.		
Factors associated with syndesmophytes at T0		
	OR (95%C.I.)	*p* value
Male sex	90.48 (1.45; 5644.87)	0.033
BMI	1.28 (0.94; 1.75)	0.118
Age at CBP onset, per year increase	1.28 (1.04; 1.58)	0.019
Constant	0 (0; 0)	0.004
X^2^ (df = 3, N = 57) 22.02, *p* < 0.001; Nagelkerke R^2^ 64.5% The model classified correctly 89.5% cases.		

Results are expressed as OR and its 95%C.I. CBP = Chronic back pain, BASDAI = Bath Ankylosing Spondylitis Disease Activity Index, MASES = Maastricht Ankylosing Spondylitis Enthesitis Score.

**Table 3:** Multivariate analysis of factors associated with mNY score at T0 and at T24, results of multiple regression analysis.

Factors Associated with mNY Score at T0 (Variables at T0)				
	B (95%C.I.)	β	t	*p* Value
Constant	2.2 (1.4; 3.1)	0	5.5	<0.001
Male sex	0.1 (−0.3; 0.5)	0.1	0.7	0.464
BASDAI at T0, per unit increase	−0.03 (−0.1; 0.02)	−0.1	−0.9	0.347
Fetuin-A at T0 (µg/mL), per 10-unit increase	−0.07 (−0.098; −0.04)	−0.5	−4.6	<0.001
Uveitis at T0	−0.6 (−1.3; 0.1)	−0.2	−1.8	0.085
R^2^ = 0.37, F (4) = 7.45; *p* < 0.001, N = 57				
Factors Associated with mNY Score at T24 (Variables at T24)				
	B (95%C.I.)	Β	t	*p* Value
Constant	1.6 (0.8; 2.4)		4.1	<0.001
BASDAI at T24, per unit increase	−0.1 (−0.2; 0.1)	−0.2	−1.2	0.2
Fetuin-A at T24 (µg/mL), per 10-unit increase	−0.030 (−0.1; −0.060)	−0.3	−2.1	<0.001
R^2^ = 0.16, F (2) = 3.51; *p* = 0.40; N = 41				
Factors associated with mNY score at T24 (variables at T0, predictors)				
	B (95%C.I.)	Β	t	*p* Value
Constant	0.2 (−0.4; 0.8)	0	0.8	0.444
BASDAI at T0	−0.03 (−0.1; 0.02)	−0.1	−1.4	0.183
mNY at T0, per unit increase	0.9 (0.7; 1.1)	0.9	11.6	<0.001
Fetuin-A at T0 (µg/mL), per 10-unit increase	−0.001 (−0.02; 0.02)	0	0.1	0.937
R^2^ = 0.85; F (5) = 40.69; *p* < 0.00; N = 41				

mNY = modified criteria of New York score, BASDAI = Bath Ankylosing Spondylitis Disease Activity Index.

## Data Availability

The dataset used and analyzed during the current study are available from the corresponding authors on reasonable request.

## Supplementary Materials

The following supporting information can be downloaded at: https://www.mdpi.com/article/10.3390/ijms24043203/s1, Table S1: Characteristics of the patients in the cohort at T0 and T24; Table S2: Characteristics of the patients in the three groups of the SPACE study at baseline on the basis of radiographic and MRI examination [3]: nr-axSpA patients without signs of sacroiliitis on MRI (nr-MRI-), nr-axSpA patients with signs of sacroiliitis on MRI (nr- MRI+) and patients with radiographic signs of sacroiliitis (r-MRI+); Table S3: Fetuin-A levels at T0 and at T24 according to categorical variables (descriptive analysis); Table S4: Association between continuous variables at T0 and T24 with Fetuin-A at T0 (*n* = 57) and at T24 (*n* = 41) (descriptive analysis); Table S5: Association between categorical variables with mNY score at T0 and at T24, results of univariate analysis; Table S6: Association between continuous variables at T0 and T24 with mNY score at T0 (*n* = 57) and at T24 (*n* = 41), results of univariate analysis.
