# Supplementary material for: Fetuin-A: A Novel Biomarker of Bone Damage in Early Axial Spondyloarthritis. Results of an Interim Analysis of the SPACE Study
Source: Int J Mol Sci. 2023 Feb 6;24(4):3203. doi: 10.3390/ijms24043203 (PMC9962253; doi:10.3390/ijms24043203)
Supplement: Supplementary file 1 [file ijms-24-03203-s001.zip › ijms-2160718-supplementary.pdf]

Supplementary materials

Fetuin-A: a novel biomarker of bone damage in early axial spondyloarthritis. Results of an interim analysis of the SPACE study

Marta Favero [1,2], Francesca Ometto [1], Elisa Belluzzi [1,3,4*], Giacomo Cozzi[1], Laura Scagnellato[1], Francesca Oliviero[1], Pietro Ruggieri[4], Andrea Doria[1], Mariagrazia Lorenzin[1], and Roberta Ramonda [1,*]

Table S1. Characteristics of the patients in the cohort at T0 and T24.

	T0	T24
**Number of individuals**	57	41
**Fetuin-A, μg/mL**	222.8 (203.3 - 251)	239.7 (199 - 275.1)
**CRP, mg/L**	2 (1 - 5)	3 (2.5 - 4)
**ESR mm/h**	17.6±15.6	11 (9.8 - 14.3)
**T0**		
**BASDAI**	4.4±2.5	1.7 (0.8 - 3.6)
**BASFI**	0.8 (0.2 - 2.3)	0.6 (0.1 - 1.6)
**BASMI**	0 (0 - 1)	0 (0 - 1)
**ASDAS**	2.5±0.8	1.2 (1 - 1.5)
**HAQ**	0.1 (0 - 0.5)	0 (0 - 0)
**MASES**	3 (1 - 5)	1 (1 - 3)
**VAS pain**	4 (1 - 6)	2 (1 - 3)
**VAS disease activity**	3 (1 - 7)	2 (1 - 3)
**Night pain**	3 (0 - 6)	1 (1 - 3)
**T0**		
**mNY**	0 (0 - 1)	0 (0 - 1)
**mSASSS**	2 (0 - 5)	3 (0 - 5)
**SPARCC spine**	2 (0 - 5)	2 (0 - 4)
**SPARCC SIJ**	3 (0 - 16)	3 (0 - 9)

Continuous variables are expressed as mean ± standard deviation or median and interquartile range, as appropriate, and categorical variables as number (%). CRP = C reactive protein, ESR = erythrocytes sedimentation rate, BASDAI = Bath Ankylosing Spondylitis Disease Activity Index, BASFI = Bath Ankylosing Spondylitis Functional Index, BASMI = Bath Ankylosing Spondylitis Metrology Index, ASDAS = Ankylosing Spondylitis Disease Activity Score, HAQ = Health Assessment Questionnaire, MASES = Maastricht Ankylosing Spondylitis Enthesitis Score, VAS = Visual Analogue Scale, mNY = modified New York criteria, mSASSS = modified Stoke Ankylosing Spondylitis Spinal Score, SPARCC = Canadian Spondyloarthritis Research Consortium, SIJ = sacroiliac joint.

Table S2. Characteristics of the patients in the three groups of the SPACE study at baseline on the basis of *radiographic and MRI examination [3]:* nr-axSpA patients without signs of sacroiliitis on MRI (nr-MRI-), nr-axSpA patients with signs of sacroiliitis on MRI (nr- MRI+) and patients with radiographic signs of sacroiliitis (r-MRI+).

	All patients	nr-axSpA MRI SIJ -	nr-axSpA MRI SIJ +	r-axSpA MRI SIJ +	p-value
Number of individuals	57	19	15	23	-
Male sex	24(42.1)	4(21.0)	7(46.6)	13(56.5)	0.06
BMI	23.5 (21.1 - 26.1)	23.5 (21.3 - 26.5)	25.2 (21.9 - 26.8)	23.3 (20.8 - 24.7)	0.64
Smoking	19(33.3)	2(10.53)	7(46.67)	10(43.48)	0.03
Age at CBP onset, years	28 (22 - 36)	32 (21.5 - 38.5)	28 (24 - 35.5)	26 (22 - 32)	0.42
CBP duration	12 (8 - 18)	12 (10 - 20)	12 (9 - 18)	11 (8 - 19)	0.55
HLA-B27 positivity	22(38.6)	3(5.8)	8(53.3)	11(47.8)	0.041
Heel enthesitis	46(80.7)	18(94.7)	12(80.0)	16(69.5)	0.12
Dactilitis	13(22.8)	6(31.6)	3(20.0)	4(17.4)	0.52
IBD	8(14)	4(21.0)	2(13.3)	2(8.7)	0.51
Psoriasis	21(36.8)	6(31.6)	7(46.7)	8(4.8)	0.90
Peripheral arthritis	24(42.1)	8(42.1)	7(46.7)	9(39.1)	0.90
Family history	28(49.1)	9(47.4)	7(46.7)	12(52.2)	0.93
Response to NSAIDs	55(96.5)	19(100.0)	14(93.3)	22(95.6)	0.55
Uveitis	4(7)	1(5.3)	3(20.0)	0(0.0)	0.06
Fetuin-A, unità di misura	222.8 (203.3 - 251)	286.3 (235.4 - 321.7)	234.3 (214.4 - 250.2)	207.9 (165.4 - 216)	<0.0001
Elevated CRP or ESR	30(52.6)	7(36.8)	10(66.7)	13(56.5)	0.19
CRP levels, mg/L	2 (1 - 5)	3 (1.5 - 4)	3 (2 - 5.5)	2 (1 - 5)	0.91
ESR levels, mm/hr	17.6±15.6	10 (7 - 16)	15 (9.5 - 26)	15 (8 - 24)	0.93
BASDAI	4.4±2.5	4.7 (2.3 - 6.6)	5.1 (3.2 - 7.4)	3.4 (1.8 - 5.3)	0.97
BASFI	0.8 (0.2 - 2.3)	1.2 (0.5 - 1.5)	1.7 (0.3 - 3.4)	0.5 (0.2 - 2.2)	0.10
BASMI	0 (0 - 1)	0 (0 - 1)	0 (0 - 2)	0 (0 - 1)	0.92
ASDAS	2.5±0.8	2.6 (1.8 - 3)	2.9 (2 - 3.3)	2.4 (1.8 - 2.9)	0.64
HAQ	0.1 (0 - 0.5)	0.4 (0.1 - 0.6)	0.1 (0 - 0.6)	0.1 (0 - 0.4)	0.20
MASES	3 (1 - 5)	3 (1.5 - 5)	5 (1.5 - 6.5)	3 (1 - 4)	0.71
VAS pain	4 (1 - 6)	5 (1.5 - 6.5)	3 (1.5 - 6.5)	4 (1 - 5)	0.96
VAS disease activity	3 (1 - 7)	5 (1 - 7)	3 (1.5 - 7.5)	3 (1 - 5)	0.63
Night pain	3 (0 - 6)	4 (0 - 6.5)	3 (0.5 - 5)	3 (0 - 6.5)	0.95
mNY at Y0	0 (0 - 1)	0 (0 - 0)	0 (0 - 0)	1 (1 - 1)	<0.01
mSASSS at T0	2 (0 - 5)	2 (0 - 5)	2 (0 - 4.5)	3 (1 - 5)	0.96
SPARCC spine	2 (0 - 5)	0 (0 - 2.5)	0 (0 - 3)	3 (0 - 7.5)	0.16
SPARCC SIJ	3 (0 - 16)	0 (0 - 2,5)	0 (0 - 3)	3 (0 - 7,5)	0.0002

Continuous variables are expressed as mean±standard deviation or median and interquartile range, as appropriate, and categorical variables as number(%). Data were compared among the three groups with Kruskal-Wallis or Chi-square or Fisher's exact test as appropriate.

CBP = Chronic back pain, HLA = human leukocyte antigen, IBD = inflammatory bowel disease, NSAIDs = Non-steroidal anti-inflammatory drugs, CRP = C reactive protein, ESR = erythrocytes sedimentation rate, BASDAI = Bath Ankylosing Spondylitis Disease Activity Index, BASFI = Bath Ankylosing Spondylitis Functional Index, BASMI = Bath Ankylosing Spondylitis Metrology Index, ASDAS = Ankylosing Spondylitis Disease Activity Score, HAQ = Health Assessment

Table S3. Fetuin-A levels at T0 and at T24 according to categorical variables *(descriptive analysis)*.

	Fetuin-A at T0			Fetuin-A at T24		
	**Not present**	**Present**	**p value**	**Not present**	**Present**	**p value**
Male sex	239.9 (207.4 - 288.8)	214.5 (202.2 - 232)	0.12	262.9 (232.5 - 285.7)	204.7 (180.1 - 245)	<0.001*
Smoking	237.1 (210.8 - 288.8)	213.4 (182.1 - 223.1)	0.01*	246.5 (199 - 285.7)	236.9 (200.4 - 255.9)	0.45
HLAB27 positivity	234.3 (202.4 - 287.6)	215.2 (207.4 - 235.2)	0.14	256.3 (213.5 - 292.1)	207.6 (194.3 - 255.9)	0.14
Heel enthesitis	207.4 (182.1 - 214.8)	232 (208.6 - 262.4)	0.03*	232.8 (195.9 - 269.6)	239.7 (201.8 - 279.4)	0.93
Dactilitis	217.7 (201.3 - 256.7)	234.3 (215.9 - 244.1)	0.28	249.5 (205.9 - 279.4)	204.5 (175.9 - 239.7)	0.11
IBD	226.9 (208.6 - 251)	202.4 (178.8 - 243.3)	0.19	243.5 (204.7 - 272.6)	211.7 (182.5 - 310.8)	0.66
Psoriasis	214.4 (189.1 - 246.8)	239.9 (215.9 - 262.4)	0.054	232.5 (197.5 - 276.9)	241.6 (204.9 - 275.1)	0.94
Peripheral arthritis	235.2 (207.9 - 286.3)	215.2 (200.2 - 237.1)	0.12	240.2 (192.6 - 270.1)	239.7 (201.8 - 286.6)	0.79
Family history	211 (199.3 - 242.6)	234.7 (215.5 - 287.6)	0.04*	227.3 (204.5 - 275.1)	243.5 (194.3 - 277.9)	0.83
Response to NSAIDs	239.9 (235.4 - 286.8)	220.8 (201.4 - 251)	0.3	236.9 (214.2 - 256)	241.6 (199 - 283.7)	0.8
Uveitis	219.4 (201.4 - 251)	242.2 (234.1 - 252.6)	0.25	245 (204.6 - 281.5)	191.5 (189 - 199.2)	0.09
Elevated CRP or ESR at T0	233.2 (209.7 - 287.6)	215.7 (201.1 - 243.1)	0.27	261.1 (211.2 - 317)	232.5 (186.5 - 259.2)	0.04*
Radiographic sacroliliitis at T0	239.9 (216.4 - 287.6)	207.9 (181.7 - 215.9)	<0.001*	261.1 (210.2 - 286.6)	207.6 (182.5 - 246.5)	0.03*
Syndesmophytes at T0	224.1 (204.4 - 256.7)	209.8 (203.3 - 249.3)	0.68	236.9 (195.9 - 270.1)	272.7 (229.1 - 301.5)	0.27

*Variables achieving a significant association with fetuin-A levels p≤0.5

Continuous variables are expressed as median and interquartile range. Data were compared between the groups with Mann-Whitney U test.

HLA = human leukocyte antigen, IBD = inflammatory bowel disease, NSAIDs = Non-steroidal anti-inflammatory drugs, CRP = C reactive protein, ESR = erythrocytes sedimentation rate.

Table S4. Association between continuous variables at T0 and T24 with Fetuin-A at T0 (n=57) and at T24 (n=41) ***(descriptive analysis).***

	All patients	Fetuin-A at T0		Fetuin-A at T24	
		Spearman's rho	p value	Spearman's rho	p value
**BMI**	23.5 (21.1 - 26.1)	0.02	0.86	0.1	0.53
**Age of onset CBP**	28 (22 - 36)	0.02	0.9	0.42	0.01*
**Duration CBP**	12 (8 - 18)	0.01	0.96	-0.04	0.8
**T0**					
**Fetuin-A, μg/mL**	222.8 (203.3 - 251)	.	.	0.3	0.06
**CRP, mg/L**	2 (1 - 5)	-0.14	0.32	0.02	0.88
**ESR mm/h**	17.6±15.6	0.13	0.32	0.15	0.35
**T24**					
**Fetuin-A, μg/mL**	239.7 (199 - 275.1)	0.3	0.06	.	.
**CRP, mg/L**	3 (2.5 - 4)	0	1	-0.01	0.95
**ESR mm/h**	11 (9.8 - 14.3)	-0.11	0.47	-0.07	0.66
**T0**					
**BASDAI**	4.4±2.5	0.2	0.15	0.18	0.25
**BASFI**	0.8 (0.2 - 2.3)	0.01	0.93	0.06	0.7
**BASMI**	0 (0 - 1)	0.09	0.52	-0.09	0.59
**ASDAS**	2.5±0.8	-0.08	0.54	-0.08	0.62
**HAQ**	0.1 (0 - 0.5)	0.07	0.58	0.1	0.53
**MASES**	3 (1 - 5)	0.2	0.13	0.11	0.47
**VAS pain**	4 (1 - 6)	0.03	0.85	0.07	0.67
**VAS disease activity**	3 (1 - 7)	0.1	0.48	0.11	0.48
**Night pain**	3 (0 - 6)	-0.14	0.31	0.25	0.12
**T24**					
**BASDAI**	1.7 (0.8 - 3.6)	0.26	0.09	0.23	0.15
**BASFI**	0.6 (0.1 - 1.6)	0.12	0.44	0.17	0.28
**BASMI**	0 (0 - 1)	0.22	0.16	0.18	0.26
**ASDAS**	1.2 (1 - 1.5)	-0.1	0.52	-0.23	0.15
**HAQ**	0 (0 - 0)	0.17	0.26	0.04	0.8
**MASES**	1 (1 - 3)	0.24	0.12	0.29	0.07
**VAS pain**	2 (1 - 3)	0.06	0.72	-0.16	0.34
**VAS disease activity**	2 (1 - 3)	0.06	0.68	0.01	0.97
**Night pain**	1.7 (0.8 - 3.6)	0.1	0.51	-0.08	0.61
**T0**					
**mNY**	0 (0 - 1)	-0.59	<0.001*	-0.38	0.01*
**mSASSS**	2 (0 - 5)	-0.06	0.65	-0.01	0.96
**SPARCC spine**	2 (0 - 5)	-0.31	0.02*	-0.31	0.05*
**SPARCC SIJ**	3 (0 - 6)	-0.25	0.06	-0.33	0.04*
**T24**					
**mNY**	0 (0 - 1)	-0.47	<0.001*	-0.39	0.01*
**mSASSS**	3 (0 - 5)	-0.08	0.62	-0.16	0.35
**SPARCC spine**	2 (0 - 4)	-0.31	0.05	-0.13	0.41
**SPARCC SIJ**	3 (0 - 9)	-0.26	0.09	-0.07	0.67

*Variables achieving a significant association with fetuin-A levels p≤0.5

Variables are expressed as mean±standard deviation or median and interquartile range, as appropriate. Associations were tested with Spearman's rho. CBP = Chronic back pain, CRP = C reactive protein, ESR = erythrocytes sedimentation rate, BASDAI = Bath Ankylosing Spondylitis Disease Activity Index, BASFI = Bath Ankylosing Spondylitis Functional Index, BASMI = Bath Ankylosing Spondylitis Metrology Index, ASDAS = Ankylosing Spondylitis Disease Activity Score, HAQ = Health Assessment Questionnaire, MASES = Maastricht Ankylosing Spondylitis Enthesitis Score, VAS = Visual Analogue Scale, mNY = modified New York criteria,

mSASSS = modified Stoke Ankylosing Spondylitis Spinal Score, SPARCC = Canadian Spondyloarthritis Research Consortium, SIJ = sacroiliac joint.

Table S5. Association between categorical variables with mNY score at T0 and at T24, results of univariate analysis.

	mNY at T0			mNY at T24		
	**Not present**	**Present**	**p value**	**Not present**	**Present**	**p value**
Male sex	0 (0 - 1)	1 (0 - 1)	0.082	0 (0 - 1)	1 (0 - 1)	0.204
Smoking	0 (0 - 1)	1 (0 - 1)	0.129	0 (0 - 1)	1 (0 - 1)	0.438
HLAB27 positivity	0 (0 - 1)	0.5 (0 - 1)	0.132	0 (0 - 1)	1 (0 - 1)	0.266
Heel enthesitis	1 (0 - 2)	0 (0 - 1)	0.017	1 (0 - 2)	0 (0 - 1)	0.19
Dactilitis	0 (0 - 1)	0 (0 - 1)	0.32	0.5 (0 - 1)	0 (0 - 1)	0.767
IBD	0 (0 - 1)	0 (0 – 0.5)	0.436	1 (0 - 1)	0 (0 - 0)	0.185
Psoriasis	0 (0 - 1)	0 (0 - 1)	0.662	0 (0 - 1)	0.5 (0 - 1)	0.879
Peripheral arthritis	0 (0 - 1)	0 (0 - 1)	0.435	1 (0 - 1)	0 (0 - 1)	0.116
Family history	0 (0 - 1)	0 (0 - 1)	0.804	0 (0 - 1)	1 (0 - 1)	0.175
Response to NSAIDs	0 (0 - 0)	0 (0 - 1)	0.881	0 (0 - 0)	1 (0 - 1)	0.35
Uveitis	0.5 (0 - 1)	0 (0 - 0.5)	0.098	0 (0 - 1)	0 (0 - 0)	0.506
Elevated CRP or ESR at T0	0 (0 - 1)	0 (0 - 1)	0.762	0 (0 - 1)	0.5 (0 - 1)	0.838
Radiographic sacroliliitis at T0	0 (0 - 0)	1 (1 - 1)	<0.001*	0 (0 - 1)	1 (1 - 2)	0.001*
Syndesmophytes at T0	0 (0 - 1)	0 (0 - 1)	0.611	1 (0 - 1)	0 (0 – 0.5)	0.285

*Variables achieving a significant association with mNY (p≤0.05)

Continuous variables are expressed as median and interquartile range. Data were compared between the groups with Mann-Whitney U test.

mNY = modified New York criteria, HLA = human leukocyte antigen, IBD = inflammatory bowel disease, NSAIDs = Non-steroidal anti-inflammatory drugs, CRP = C reactive protein, ESR = erythrocytes sedimentation rate.

Table S6. Association between continuous variables at T0 and T24 with mNY score at T0 (n=57)and at T24 (n=41), results of univariate analysis.

	All patients	mNY at T0		mNY at T24	
		Spearman's rho	p value	Spearman's rho	p value
BMI	23.5 (21.1 - 26.1)	-0.14	0.29	-0.19	0.24
Age of onset CBP	28 (22 - 36)	-0.21	0.11	-0.16	0.33
Duration CBP	12 (8 - 18)	-0.03	0.83	0.1	0.54
**T0**					
Fetuin-A, μg/mL	222.8 (203.3 - 251)	-0.59	<0.001*	-0.47	<0.001‡
CRP, mg/L	2 (1 - 5)	-0.03	0.85	0.08	0.63
ESR mm/h	17.6±15.6	-0	1	0.04	0.78
**T24**					
Fetuin-A, ng/mL	239.7 (199 - 275.1)	-0.38	0.01*	-0.39	0.01†
CRP, mg/L	3 (2.5 - 4)	0.15	0.33	0.1	0.54
ESR mm/h	11 (9.8 - 14.3)	0.11	0.47	0.15	0.35
**T0**					
BASDAI	4.4±2.5	-0.22	0.09*	-0.44	<0.001‡
BASFI	0.8 (0.2 - 2.3)	-0.11	0.41	-0.25	0.11
BASMI	0 (0 - 1)	-0.06	0.68	-0.12	0.44
ASDAS	2.5±0.8	-0.08	0.55	-0.3	0.06
HAQ	0.1 (0 - 0.5)	-0.13	0.33	-0.16	0.31
MASES	3 (1 - 5)	-0.21	0.11	-0.22	0.17
VAS pain	4 (1 - 6)	-0.13	0.35	-0.42	0.01‡
VAS disease activity	3 (1 - 7)	-0.14	0.29	-0.41	0.01‡
Night pain	3 (0 - 6)	-0	0.99	-0.25	0.12
**T24**					
BASDAI	1.7 (0.8 - 3.6)	-0.25	0.11	-0.28	0.07†
BASFI	0.6 (0.1 - 1.6)	-0.07	0.64	-0.17	0.28
BASMI	0 (0 - 1)	-0.03	0.87	-0.1	0.53
ASDAS	1.2 (1 - 1.5)	0.1	0.55	0.19	0.23
HAQ	0 (0 - 0)	-0.08	0.63	-0.13	0.42
MASES	1 (1 - 3)	-0.17	0.27	-0.16	0.32
VAS pain	2 (1 - 3)	0.01	0.97	-0.15	0.35
VAS disease activity	2 (1 - 3)	0.04	0.8	-0.12	0.44
Night pain	1.7 (0.8 - 3.6)	0.02	0.89	-0.12	0.46
**T0**					
mNY	0 (0 - 1)	.	.	0.89	<0.001‡
mSASSS	2 (0 - 5)	0.04	0.79	0.07	0.66
SPARCC spine	2 (0 - 5)	0.30	0.02§	0.27	0.091‡
SPARCC SIJ	3 (0 - 6)	0.42	<0.001§	0.437	<0.001‡
**T24**					
mNY	0 (0 - 1)	0.89	<0.001	.	.
mSASSS	3 (0 - 5)	0.2	0.2	0.2	0.22
SPARCC spine	2 (0 - 4)	0.24	0.13	0.29	0.07§
SPARCC SIJ	3 (0 - 9)	0.33	0.03	0.38	0.01§

*Variables (at T0) included in the multivariate analysis as achieving an association with mNY at T0 with p<0.1
†Variables (at T24) included in the multivariate analysis as achieving an association with mNY at T24 with p<0.1
‡Variables (at T0) included in the multivariate analysis as achieving an association with mNY at T24 with p<0.1 (potential predictors)

§Variables achieving a significant association with the outcome, not included in the multivariate analysis (expected association between radiographic measures)

Variables are expressed as mean±standard deviation or median and interquartile range, as appropriate. Associations were tested with Spearman's rho.

CRP = C reactive protein, ESR = erythrocytes sedimentation rate, BASDAI = Bath Ankylosing Spondylitis Disease Activity Index, BASFI = Bath Ankylosing Spondylitis Functional Index, BASMI = Bath Ankylosing Spondylitis Metrology Index, ASDAS = Ankylosing Spondylitis Disease Activity Score, HAQ = Health Assessment Questionnaire, MASES = Maastricht Ankylosing Spondylitis Enthesitis Score, VAS = Visual Analogue Scale, mNY = modified New York criteria, mSASSS = modified Stoke Ankylosing Spondylitis Spinal Score, SPARCC = Canadian Spondyloarthritis Research Consortium, SIJ = sacroiliac joint.
